# Protein Electrophoresis and Haptoglobin Values for Captive Bongo (*Tragelaphus eurycerus*)

**DOI:** 10.3389/fvets.2021.646500

**Published:** 2021-04-28

**Authors:** Susan L. Bartlett, Nadine Lamberski, Kristopher L. Arheart, Carolyn Cray

**Affiliations:** ^1^Wildlife Conservation Society, Zoological Health Program, Bronx, NY, United States; ^2^San Diego Zoo Safari Park, Escondido, CA, United States; ^3^Division of Biostatistics, Department of Public Health Sciences, University of Miami Miller School of Medicine, Miami, FL, United States; ^4^Division of Comparative Pathology, University of Miami Miller School of Medicine, Miami, FL, United States

**Keywords:** acute phase protein, antelope, bongo, bovid, inflammation, reference interval

## Abstract

Serum samples collected from 37 clinically normal bongo (*Tragelaphus eurycerus*) and 13 abnormal bongo were tested using assays for acute-phase proteins (APPs) and by protein electrophoresis. Abnormal bongo samples (*n* = 27) had significantly higher levels of fibrinogen (FIB) (*p* < 0.001) and trending but not significantly increased haptoglobin (HP) (*p* = 0.07) vs. samples from normal bongo (*n* = 37). There were no significant differences in values for total white blood cell counts or for any of the fractions determined by protein electrophoresis. Clinically normal female bongo (*n* = 19) had significantly lower levels of FIB than normal males (*n* = 18) (*p* = 0.014), and this observation was also made with samples from the clinically abnormal group (*p* = 0.004). Many weak to moderate significant correlations were observed with increasing age, including increased globulins, FIB, and HP and decreased albumin-to-globulin (A/G) ratio and albumin. In clinical cases reviewed in this study, mild HP changes categorized this reactant as a minor APP, which contrasts with the major APP classification of HP in the related species of the cow. The preliminary data indicate that the quantitation of these APPs may offer value in assessing inflammation in this species, but additional studies are needed.

## Introduction

The acute-phase response is a key part of the innate immune system involving the expression of acute-phase proteins (APPs), which are recognized as biomarkers of inflammation (1). Quantitation of APPs has been demonstrated to aid in the detection and prognostication of various diseases and inflammatory conditions in companion and large animals (2–4). In addition, APP assays have been reported to be important tools in the clinical management of the rhino, manatee, zebra, and elephant among other non-domesticated mammals (5–9).

APPs can be evaluated in the clinical laboratory using serum protein electrophoresis methods and specific assays for individual proteins. The protein electrophoretogram is believed to reflect over 200 APPs *via* the migration of different globulin fractions as well as the valid quantitation of albumin (10). Whereas albumin is considered a negative APP based on decreased levels during inflammatory processes, positive APP may be categorized by the level and timeline of increase (1, 2). Major APPs increase by 100–1,000-fold within 48 h and decline rapidly during recovery. Moderate APPs increase 5–10-fold within 2–3 days and decline more slowly. Lastly, minor APPs increase by 50–100% of baseline. The specific APPs and the magnitude of the increase vary depending on the species, necessitating basic investigations to evaluate methods and obtain evidence-based validation of APP assays (2, 11).

There are two subspecies of bongo: mountain or eastern bongo (*Tragelaphus eurycerus isaaci*) and the lowland or western bongo (*Tragelaphus eurycerus eurycerus*). These members reside mainly in the forests of west and central Africa and are considered near-threatened in the wild by the International Union for Conservation of Nature (IUCN) (12). Upon review of necropsy records from 96 captive bongos, both infectious causes of death inclusive of bacteria and fungi and chronic conditions inclusive of amyloidosis, neoplasia, and emaciation were identified (13). The bongo is a member of the family Bovidae. Although mammals including cattle with acute inflammation may exhibit a brief leukopenia, within days, this generally resolves, and only an increase in the neutrophil/lymphocyte ratio may remain with a left shift (14). Therefore, assessing the white blood cell (WBC) count for an indication of inflammation or infection in cattle may be insufficient. Assessing the APP response as well as performing serum protein electrophoresis has been demonstrated to characterize the inflammatory response in cattle (15). Notably, haptoglobin (HP) has been described to be a major APP in the cow (2, 16). Thus, the goals of the present study were two-fold. First, to establish preliminary reference intervals (RIs) for fractions quantitated by electrophoresis as well as HP levels. Second, to evaluate both tools vs. other markers of inflammation in clinically normal vs. abnormal bongo.

## Materials and Methods

Serum samples were obtained from clinically normal captive adult bongos (*n* = 37) from four institutions within the United States accredited by the Association of Zoos and Aquariums. The study population consisted of 19 females and 18 males ranging in age from 1 to 13 years. A total of 27 samples were also collected from 13 animals (seven males and six females; age range 1–16 years) that were determined to be clinically abnormal. Complete blood cell counts were performed by the respective institutions on all but nine samples. Fibrinogen (FIB) was performed on samples from 17 clinically normal animals and 10 clinically abnormal bongos by the respective institutions. Analysis of abnormal individuals included cases of amyloidosis, meningitis/endocarditis, abscess, pneumonia, metritis, osteomyelitis, traumatic fetlock subluxation, and a gore wound. As this was a study composed mostly of banked samples obtained over a 15-year period and involving several institutions, there was no control provided to animal handling for sampling, site of blood sampling, and sample preparation. Non-lipemic, non-hemolyzed serum samples were stored at −80°C or shipped on ice on the day of collection to the University of Miami (Miami, FL, USA).

Protein electrophoresis was conducted as previously described using Helena split beta gels (Helena Laboratories, Beaumont, TX, USA) (9). Fraction delimits were placed using conventions established for mammals. HP testing was conducted as previously described on the Rx Daytona analyzer (Randox Laboratories, Kearneysville, WV, USA) (9). Linear regression analysis using an abnormal bongo sample showed that this assay did not deviate from linearity.

RIs were calculated per ASVCP guidelines (17). Outliers were identified by Tukey analysis but not removed. Most of the analyte results were non-Gaussian in distribution, as determined by the D'Agostino-Pearson test. The robust method was used with Box–Cox transformation (for non-Gaussian data) using MedCalc software 18.11.3 (Ostend, Belgium). The lambda for the transformation was acquired from the data as an option in the software. For comparison of data from clinically normal vs. abnormal bongo, as the data were not normally distributed, a general linear mixed model was used to analyze the data. A link for a Poisson distribution was used. The fixed effects were group (normal vs. abnormal), sex (male vs. female), and age plus group × sex, group × age, and sex × age interactions. A backward elimination of effects that were not significant was performed to simplify the model, resulting in a model including only group. Some animals were measured several times; therefore, a random effect of animal nested within the group was included in the model to control for the variance due to the repeated measurements. A variance components covariance matrix was used to represent the correlated data structure. The model means, standard errors, and a *p*-value for a comparison of the group means are reported. The data are reported as model means and standard errors. SAS Version 9.4 (SAS Institute, Inc., Cary, NC) was used for all analyses.

## Results

**Figure 1:**
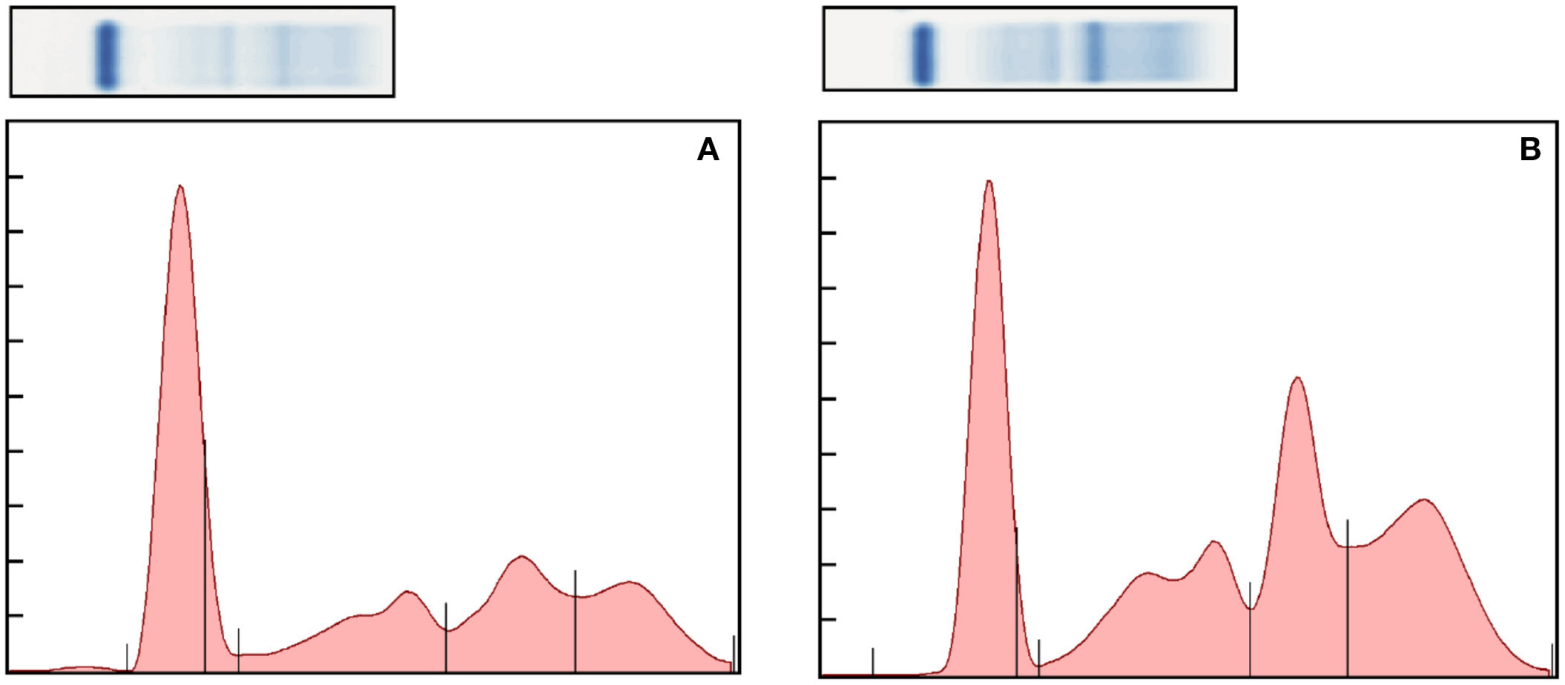
Representative normal **(A)** and abnormal **(B)** electrophoretograms of the bongo. The fractions, from left to right, are albumin, alpha 1, alpha 2, beta, and gamma globulins. The A/G ratio of the electorphoretograms is 0.69 and 0.36, respectively.

Preliminary RIs were generated for electrophoresis fractions and HP (Table 1). Representative normal and abnormal electrophoretograms are presented in Figure 1. There were significant positive correlations with increasing age and total protein (*r* = 0.41, *p* = 0.0004), alpha 2 globulins (*r* = 0.34, *p* = 0.0036), beta globulins (*r* = 0.37, *p* = 0.0015), and gamma globulins (*r* = 0.27, *p* = 0.0221). There were significant negative correlations with increasing age and albumin (*r* = −0.31, *p* = 0.0095) and albumin-to-globulin (A/G) ratio (*r* = −0.57, *p* < 0.001). There was also a positive correlation with increasing age and levels of HP (*r* = 0.32, *p* = 0.0064) and FIB (*r* = 0.37, *p* = 0.017).

**Table 1:** Reference intervals (RIs) for serum protein electrophoresis fractions and haptoglobin for 37 normal adult captive bongos (*Tragelaphus eurycerus*).

**Analyte**	**Mean**	**SD**	**Median**	**Min–Max**	**RI**	**LRL^**b**^90% CI**	**URL^**b**^ 90% CI**	**Distribution^**c**^**
Total protein, g/dl	7.0	1.1	6.8	5.4–10.2	4.5–9.1	3.9–5.2	8.4–9.8	NG
A/G ratio	0.66	0.19	0.64	0.28–1.05	0.26–1.06	0.18–0.35	0.96–1.14	G
Albumin, g/dl	2.71	0.48	2.72	1.31–3.76	1.77–3.75	1.52–2.04	3.45–4.00	G
Alpha 1 globulins, g/dl	0.30	0.14	0.27	0.17–0.91	0.03–0.56	0–0.10	0.42–0.68	NG
Alpha 2 globulins, g/dl	0.82	0.24	0.78	0.49–1.79	0.27–1.27	0.09–0.47	1.07–1.45	NG
Beta globulins, g/dl	0.89	0.27	0.86	0.54–1.75	0.30–1.40	0.14–0.43	1.21–1.57	NG
Gamma globulins, g/dl	2.31	0.82	2.12	1.07–4.09	0.55–4.00	0.26–0.90	3.52–4.36	G
Haptoglobin, mg/ml	0.68	0.44	0.66	0.12–1.44	0.10–1.60^a^	0^c^	1.39–1.78	NG

*The robust method using Box–Cox transformed data was used for all RI calculations involving NG data*.

a *Lower limit of detection is 0.1 mg/ml*.

b *LRL, lower reference limit; URL, upper reference limit*.

c *LRL was below 0*.

c *NG, non-Gaussian; G, Gaussian; lambda for Box–Cox transformation: total protein, −1.72; alpha 1 globulins, −1.23; alpha 2 globulins, −0.84; beta globulins, −0.69; and haptoglobin, 0.42*.

APP values and electrophoresis fractions were also examined in clinically abnormal bongos (Table 2). FIB (*p* < 0.001) was significantly higher in the abnormal animals (*n* = 13). There were no significant differences in values for total WBC counts or for any globulin fraction. HP levels were not significantly different between the clinical groups (*p* = 0.07). Normal females (*n* = 19) had significantly lower levels of FIB than males (*n* = 18): 259 ± 93 mg/dl vs. 469 ± 84 mg/dl (*p* = 0.014). This was also observed with clinically abnormal animals: female 534 ± 96 mg/dl (*n* = 6) vs. male 865 ± 67 mg/dl (*n* = 7, *p* = 0.014). Clinically abnormal females also had a lower albumin than abnormal males (2.13 ± 0.17 vs. 2.40 ± 0.19, *p* = 0.011).

**Table 2:** Mean, standard error (SE), and significance of results of serum acute-phase proteins, protein electrophoresis fractions, and total white blood cell (WBC) for 37 normal adult captive bongos (*Tragelaphus eurycerus*) (37 samples) and 13 abnormal bongos (27 samples).

**Analyte**	**Normal bongo mean ± SE**	**Abnormal bongo mean ± SE**	***P*-value**
Total protein, g/dl	6.8 ± 0.4	7.6 ± 0.5	0.26
A/G ratio	0.69 ± 0.14	0.43 ± 0.13	0.22
Albumin, g/dl	2.67 ± 0.27	2.17 ± 0.28	0.23
Alpha 1 globulins, g/dl	0.24 ± 0.08	0.20 ± 0.09	0.75
Alpha 2 globulins, g/dl	0.96 ± 0.16	1.41 ± 0.23	0.12
Beta globulins, g/dl	1.04 ± 0.17	1.54 ± 0.24	0.10
Gamma globulins, g/dl	1.84 ± 0.23	2.23 ± 0.29	0.30
Haptoglobin, mg/ml	0.65 ± 0.13	1.10 ± 0.20	0.07
Fibrinogen, mg/dl	342 ± 30	525 ± 48	<0.001
WBC (10^3^/μl)	6.3 ± 0.5	7.8 ± 0.9	0.17

Analysis of abnormal individuals included cases of meningitis/endocarditis, abscess, pneumonia, metritis, osteomyelitis, traumatic fetlock subluxation, and a gore wound (Table 3). FIB was consistently observed to be increased across all cases, but HP was increased mildly, and albumin was decreased in two cases.

**Table 3:** Acute-phase protein and protein electrophoresis fractions in clinically abnormal captive bongos (*Tragelaphus eurycerus*).

**Analyte**	**1 Meningitis endocarditis**	**2 Abscess**	**3 Pneumonia**	**4 Metritis**	**5 Osteomyelitis**	**6Subluxation**	**7 Gore wound**	**RI**
HP, mg/ml	0.3	1.00	1.37	1.06	**1.77**	**1.85**	1.11	0.10–1.60
Fibrinogen, mg/dl	**600**	NP^a^	**600**	**1,000**	**1,000**	**500**	**1,200**	117 ± 228^b^
Total protein, g/dl	8.6	6.4	8.0	5.4	8.2	8.3	5.2	4.5–9.1
A/G ratio	0.40	0.47	0.28	0.50	0.74	0.67	0.36	0.26–1.06
Albumin, g/dl	2.42	2.06	**1.75**	1.79	3.49	3.33	**1.37**	1.77–3.75
Alpha 1 globulins, g/dl	0.34	0.39	0.43	0.23	0.41	0.24	0.18	0.03–0.56
Alpha 2 globulins, g/dl	0.68	0.85	**1.49**	0.68	0.93	0.82	0.60	0.27–1.27
Beta globulins, g/dl	**2.35**	0.61	0.86	0.64	0.83	1.31	0.48	0.30–1.40
Gamma globulins, g/dl	2.77	2.49	3.46	2.06	2.53	2.60	2.57	0.55–4.00
WBC (10^3^/μl)	6.7	NP^a^	4.8	**28.2**	6.3	6.6	**11.2**	5.8 ± 2.1^b^

*Values in bold are outside the reference interval (RI)*.

a *NP, not performed*.

b *Reference range from International Species Information System. Reference ranges for physiologic values in captive wildlife [CD-ROM]. Apple Valley (MN): International Species Information System; c2002., presented as mean ± SE*.

## Discussion

This is the first report of protein electrophoresis in the bongo; fraction migration was found to be similar to other mammals including the cow, a related species (10, 15). While RIs using American Society for Veterinary Clinical Pathology (ASVCP) guidelines for electrophoresis are not available for other antelope species, the current results demonstrated mostly overlapping or similar levels of alpha 1, alpha 2, and beta globulins but higher gamma globulins than domestic cattle (cattle mean values: alpha 1, 0.58 g/dl; alpha 2, 0.58 g/dl; beta, 0.75 g/dl; gamma, 1.67 g/dl) (15). Compared to white-tailed deer (*Odocoileus virginianus*), a lower A/G ratio (vs. mean 1.25), lower albumin (vs. mean 3.90 g/dl), and higher gamma globulins (vs. mean 0.72 g/dl) were observed in the bongo (18). Interestingly, a varying level of gamma globulins in normal bongo at different institutions was observed (data not shown). It is possible that this difference as well as those vs. cattle and deer was related, in part, to different husbandry/management, genetic predisposition, or subclinical illness.

Protein electrophoresis provides a broad picture of the acute-phase response with potential increases in globulins (representing the positive APP and immunoglobulins) and decreases in albumin. Albumin is classified as a negative APP; decreases are believed to be related to increased oxidation and scavenging, so that synthesis of other proteins important in the inflammatory process can be upregulated (19). Notably, no significant differences were found in protein fractions between the clinically normal and abnormal groups. This may be related to the diversity of diseases and conditions but also using single measures obtained during planned diagnostic procedures. The relative insensitivity of protein electrophoresis vs. specific APP assays has previously been reported in non-domesticated mammals and horses (6, 9, 20). However, in dairy cows with hoof disease, mastitis, and postpartum metritis, significant decreases in albumin and increases in alpha 1 and beta globulins were observed (21). When examining representative clinical cases in the bongo, a few increases in globulins were observed, which may represent changes in major APP levels. Only two of seven cases showed a decrease in albumin; the case of the gore wound seemed to have the most pronounced changes across several measures of inflammation. Additional studies should be undertaken, examining protein electrophoresis in animals with more severe inflammatory processes.

HP RIs in healthy bongos in this study were 0.10–1.60 mg/ml. HP was also confirmed as a major APP in the water buffalo, with normal levels reported as 0.13 ± 0.01 mg/ml (22). Although HP has been detected in impala using a colorimetric assay, RIs were not reported (8). In a study of sarcoptic mange in the ibex, HP was reported as a minor APP with normal levels of 0.58 ± 0.09 mg/ml (23). In healthy domestic cattle, HP was undetectable, but increases of 100-fold in clinically abnormal cattle support HP as a major APP (2, 24). Low, undetectable levels of APP in normal animals are a hallmark of major APP, whereas minor APPs have a constitutive expression that results in detectable levels in normal animals (1).

Although HP has been observed to increase with a variety of diseases in cattle, elevated expression in clinically abnormal bongos was not consistently observed (22). As samples from a variety of clinical presentations were examined, it appears that HP is a minor APP in this species, with less than a two-fold increase in expression. Samples from four cases of amyloidosis were also reviewed (data not shown). Repeated measures over a period of a few months for two cases showed inconsistent increases in HP levels. In contrast, HP (as a major APP) was increased in 88% of cows with amyloidosis (24). In total, these data support that HP in the bongo is a minor APP. HP is often characterized in other species, when a minor APP, as a better marker of chronic inflammation (1). Additional studies should be undertaken to examine samples from clinically abnormal bongos with more diverse clinical presentations to confirm this categorization.

FIB levels in the normal bongo group (342 ± 30 mg/dl) were comparable to Arabian oryx (291 ± 82 mg/dl), impala (250 ± 18 mg/dl), and blackbuck (395 ± 50 mg/dl) (25). Normal females had lower levels of FIB than males, and this difference was also observed in the clinically abnormal cohort group. Reasons for this difference may include a higher risk of inflammation or infection in males, perhaps due to increased traumatic injuries if kept with other males, or other stressors. Age-related changes included increases in total protein and globulins, while albumin declines have been observed in other species (10). An age-associated increase in inflammatory markers is seen in humans and, while the exact mechanism is unknown, is suspected to be related to changes in adiposity, sex hormones, and oxidative damage (26).

The results reflect changes consistent with positive and negative APPs in other mammals (1). Positive APPs may be expressed constitutively and increase in varying magnitude with an acute-phase response. Consistent with classifications in other mammals, FIB and HP were positive APPs in the bongo. In the present study, in comparison of normal and abnormal bongos by group, only changes in FIB were statistically significant. In addition, six of six clinical cases showed elevated FIB. This included a diverse presentation inclusive of various infections and trauma. While WBC counts were elevated in two bongos with high FIB, the significant changes in FIB contrast the lack of consistent elevation in total WBC. The poor correlation between WBC values and APP has been reported in other species with the understanding that these are independent measures of the acute-phase response with different timelines of expression (10).

The RI produced from this study should be considered preliminary, as the guidelines for RI generation presented by the ASVCP could not be adhered to throughout the study (17). As this was a multi-institutional study utilizing samples collected over an approximately 15-year time span, there were differences in sample collection (including method of animal restraint, venipuncture location, and technique) and processing [sample preparation and varied implementation of FIB and complete blood cell (CBC) testing] that could potentially have influenced the results. To this point, stress-associated changes in hematological values and iron but not HP were reported in free-ranging white rhinoceros subject to transport with chemical immobilization (27). For the most part, HP is found to be stable with prolonged freezing (28).

In the present study, it was noted that the calculated RIs for A/G ratio and gamma globulins were broad, and the upper RI for HP was high vs. similar species, although these animals were determined to be clinically normal during the study period. It is possible that animals with underlying infectious/inflammatory conditions were in the presumed normal population. To this point, as amyloidosis and other chronic conditions are a common issue in the bongo, some of these differences observed in the current study could reflect early disease (13). Overall, however, it is proposed that APPs are not only important biomarkers of inflammation that have a key prognostic value but that they also are tools in general health assessments with the goal of detecting possible subclinical disease or conditions (1, 2). In the bongo, the use of FIB, HP, and albumin can, at minimum, provide a basic evaluation of underlying inflammation and be used as adjunct tools to traditional hematological measures. Further studies should be conducted identifying major APPs, developing bongo-specific APP reagents, and obtaining repeated measures on a larger sample set of normal and abnormal animals with controlled sample acquisition and processing to better address the utility of APP testing in this species.

## Data Availability Statement

The raw data supporting the conclusions of this article will be made available by the authors, without undue reservation.

## Ethics Statement

Ethical review and approval was not required for the animal study because the samples were collected as part of routine or clinical health assessments. These were banked samples.

## Author Contributions

SB and CC shared in designing the study. CC performed the data collection. CC, SB, and KA performed data analysis. SB, CC, KA, and NL wrote and revised the manuscript. All authors contributed to the article and approved the submitted version.

## Conflict of Interest

The authors declare that the research conducted in the absence of any commercial or financial relationships that could be construed as a potential conflict of interest.
